# Successful Rescue Treatment for Acute Ischemic Stroke Due to Intracranial Atherosclerotic Disease Using the Neuroform Atlas Stent

**DOI:** 10.7759/cureus.78569

**Published:** 2025-02-05

**Authors:** Yuta Oka, Yoshinobu Horio, Jota Tega, Koichiro Takemoto, Hiroshi Abe

**Affiliations:** 1 Neurosurgery, Fukuoka Seisyukai Hospital, Fukuoka, JPN; 2 Neurosurgery, Fukuoka University Hospital, Fukuoka, JPN

**Keywords:** atherosclerosis, endovascular surgery, neuroform atlas, rescue stenting, thrombectomy

## Abstract

The recanalization rate of endovascular therapy for acute ischemic stroke in intracranial atherosclerotic disease (ICAD) is inferior to that of cardiogenic stroke. This case study presents the long-term outcomes of a patient who underwent implantation of the Neuroform Atlas stent for the treatment of intracranial carotid artery stenosis, despite repeated re-occlusions after percutaneous transluminal angioplasty (PTA). A 75-year-old woman was transferred to our hospital with aphasia. Diffusion-weighted imaging revealed the absence of recent infarction. Magnetic resonance angiography indicated an occlusion of the left internal carotid artery (ICA). Diagnostic cerebral angiography revealed severe stenosis of the left ICA, accompanied by a markedly delayed blood flow to the left middle cerebral artery region. Therefore, we decided to proceed with endovascular surgery. The guide catheter was advanced into the ICA which was spontaneously recanalized. The patient again exhibited symptoms of aphasia the next day. We decided to perform PTA, but restenosis was observed immediately. The Neuroform Atlas was placed within the stenosis. Following this, the ICA was confirmed to be open. At the one-year follow-up, cerebral angiography demonstrated optimal patency of the left ICA. The Neuroform Atlas demonstrated favorable angiographic patency over one year when implanted as a rescue stent in revascularization procedures for ICAD. Hence, its use for rescue purposes in acute settings may be considered a treatment option.

## Introduction

The recanalization rate of endovascular therapy (EVT) for acute ischemic stroke (AIS) due to intracranial atherosclerotic disease (ICAD) is inferior to that observed in cases of cardiogenic stroke. EVT techniques, including contact aspiration, stent retrieval, and percutaneous transluminal angioplasty (PTA), have been utilized in the management of ischemic stroke due to ICAD. However, these approaches frequently prove to be refractory to treatment [1]. In cases of ischemic stroke due to ICAD that are refractory to conventional recanalization therapy, alternative treatment options are required. One potential treatment option is the placement of an intracranial stent, such as the Neuroform Atlas off-label use. While there have been several reports of the use of the Neuroform Atlas for rescue stenting, there have been no reports of long-term follow-up imaging [2,3]. This case study presents the case of a patient who underwent stenting with the Neuroform Atlas due to an AIS caused by severe intracranial carotid artery stenosis with recurrent stenosis. At the one-year follow-up, the patient exhibited a favorable outcome, with no evidence of restenosis on cerebral angiography.

## Case presentation

**Figure 1:**
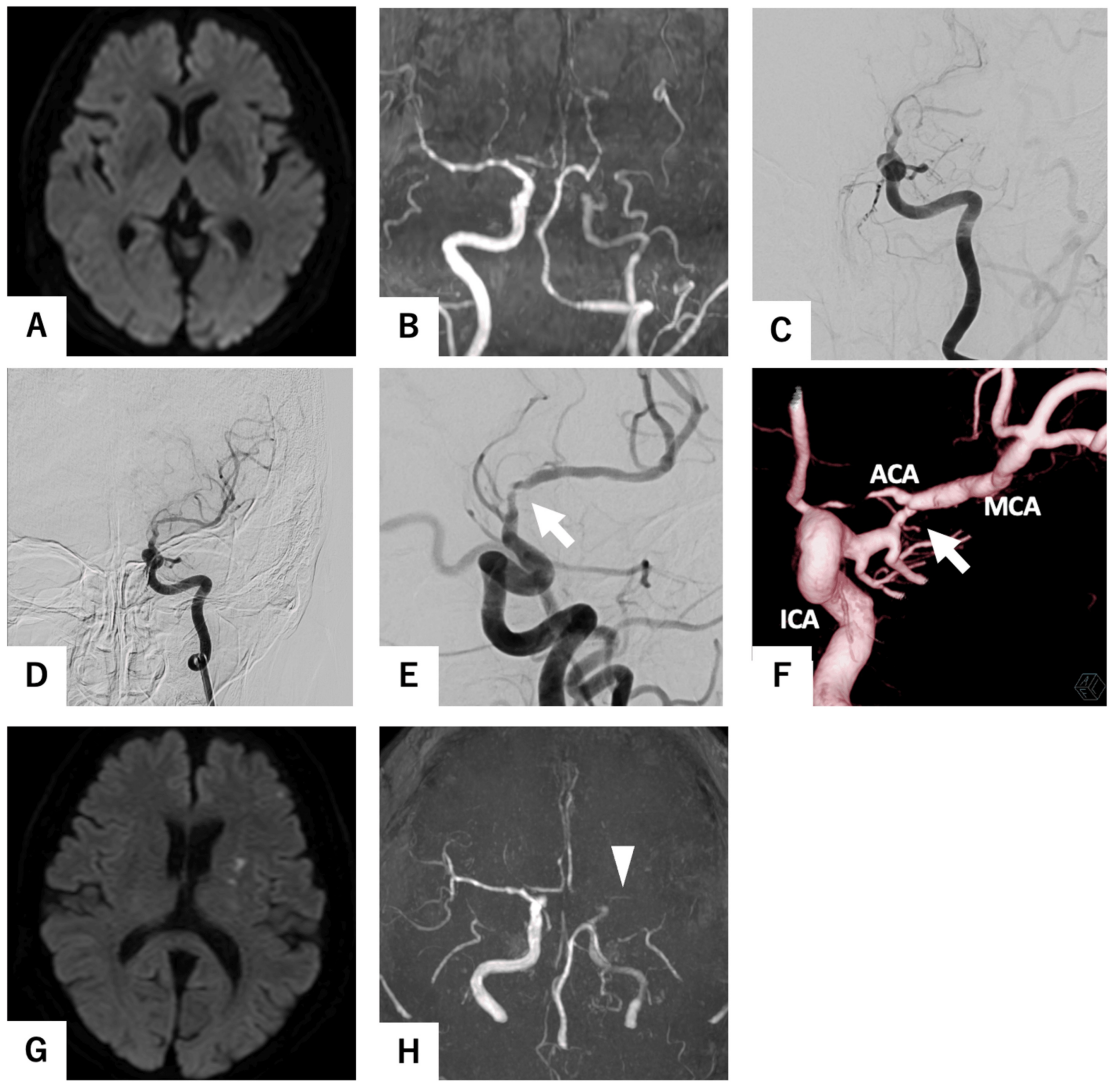
Initial magnetic resonance imaging (MRI), initial cerebral angiography, and MRI on the following day. (A) No high-intensity area was found on the diffusion-weighted image (DWI). (B) Magnetic resonance angiography (MRA) demonstrated an occlusion of the left internal carotid artery (ICA). (C) The initial ICA angiogram revealed a severe stenosis of the left ICA, accompanied by a markedly delayed blood flow to the left middle cerebral artery (MCA) region. (D) The guide catheter was advanced into the ICA and the ICA was spontaneously recanalized. (E) The stenosis remained at the C1 portion (white arrow). (F) A three-dimensional rotational angiogram demonstrated that the stenosis (Warfarin-Aspirin Symptomatic Intracranial Disease (WASID) 55%) was present at the C1 portion (white arrow). (G) The following day, DWI revealed new infarcts in the left basal ganglia and left frontal lobe. (H) MRA revealed a high-signal area in the left MCA (white arrowhead).

**Figure 2:**
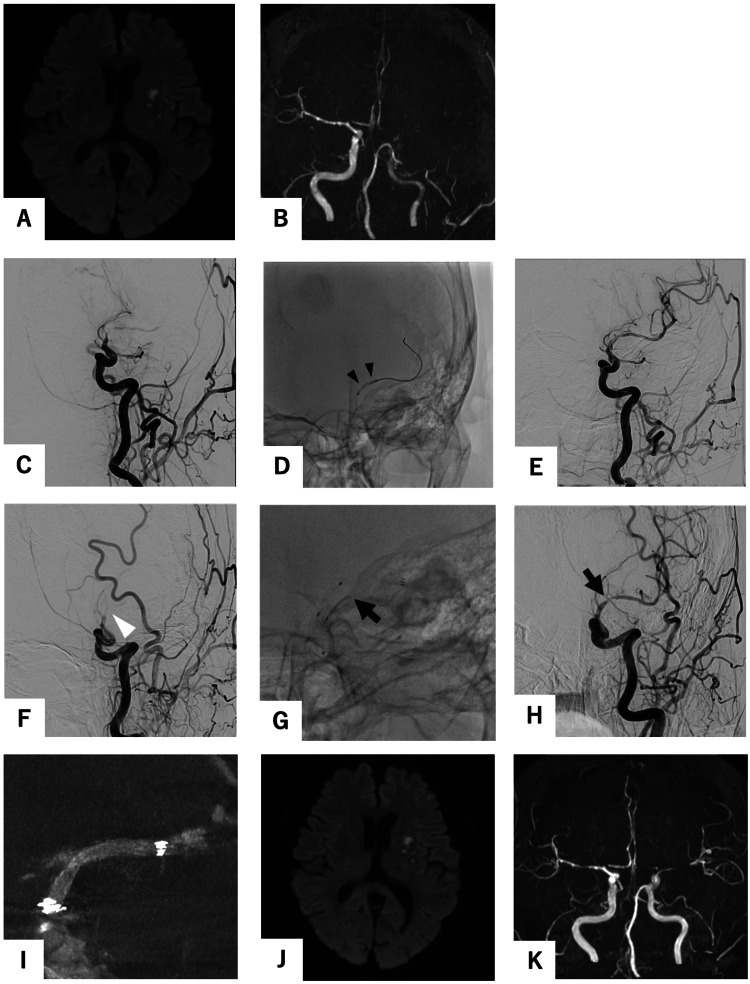
Endovascular surgery and postoperative magnetic resonance imaging (MRI). (A) Diffusion-weighted imaging (DWI) at the time of deterioration showed no new infraction area compared to the previous one. (B) Magnetic resonance angiography (MRA) showed a re-occlusion of the left ICA. (C) As in the first cerebral angiogram, common carotid artery angiogram (CCAG) revealed a severe stenosis of the left ICA, accompanied by a markedly delayed blood flow to the left middle cerebral artery region. (D) A Gateway MR 2.0 × 12 mm (Stryker, Kalamazoo, MI, USA) (black arrowhead) was introduced into the stenotic lesion. Despite performing percutaneous transluminal angioplasty (PTA) (E), restenosis was observed immediately (white arrowhead). (F) We performed PTA using Gateway MR 2.0 × 12 mm at the stenotic lesion (black arrowhead) after PTA allowed spontaneous recanalization. Soon after, CCAG showed restenosis at balloon expansion segment (white arrowhead). (G) The Neuroform Atlas 3.0 × 21 mm (Stryker, Kalamazoo, MI, USA) (black arrow) was placed within the stenosis. (H) Subsequent to the stenting procedure, the CCAG demonstrated the existence of recanalization (black arrow), exhibiting no indications of delayed blood flow. (I) Cone-beam computed tomography showed that the Neuroform Atlas was well adhered to the vessel wall and the stenosis was dilated. (J) Postoperative DWI showed no enlargement of the infraction area. (K) Postoperative MRA showed more improved intensity in the peripheral left middle cerebral artery.

A 75-year-old woman was referred to our hospital while receiving intravenous recombinant tissue plasminogen activator (rt-PA) after magnetic resonance angiography (MRA) revealed an occlusion of the left internal carotid artery (ICA) (Figures 1A, 1B). She had been diagnosed with aphasia and was under the care of her primary physician. She had hypertension and dyslipidemia, but no atrial fibrillation was found on ECG at the time of the initial examination. Diffusion-weighted imaging (DWI) did not indicate the presence of a new infarction. Upon arrival, motor aphasia and dysarthria were observed, with a National Institutes of Health Stroke Scale score of 5. The diagnostic cerebral angiography revealed a severe stenosis of the left ICA (C1) (Figure 1C), accompanied by a markedly delayed blood flow to the left middle cerebral artery (MCA) region. Therefore, we decided to proceed with endovascular surgery. The guide catheter was advanced into the left ICA, and the ICA was spontaneously recanalized (Figure 1D). Three-dimensional rotational angiography (3DRA) revealed residual severe stenosis of the left ICA distal to the anterior choroid artery, with a Warfarin-Aspirin Symptomatic Intracranial Disease (WASID) of 55% stenosis [4] (Figures 1E, 1F). Aphasia demonstrated an immediate improvement following the recanalization of the ICA. The following morning, 24 hours after the administration of rt-PA, a regimen of aspirin 200 mg, prasugrel 3.75 mg, and heparin 10,000 units per day was initiated. On the evening in question, the patient again exhibited symptoms of aphasia. Furthermore, DWI demonstrated an increased infarction area (Figure 2A), while MRA exhibited hypointensity of the left ICA (Figure 2B). Accordingly, we decided to perform PTA following the administration of prasugrel 20 mg.

Endovascular procedure

Endovascular surgery was performed under local anesthesia. An 8-F long sheath was inserted into the right femoral artery. A bolus of heparin (5,000 units) was administered initially. An OPTIMO 8F (Tokai Medical Products, Kasugai, Aichi, Japan) was then guided into the left ICA. A Gateway MR 2.0 × 12 mm (Stryker, Kalamazoo, MI, USA) was introduced into the stenotic lesion. We performed PTA using Gateway MR 2.0 × 12 mm at 4.7 atm for seven seconds, shifted the position, and performed a second PTA at 3.4 atm for 25 seconds. Despite performing PTA, restenosis was observed immediately (Figures 2D-2F). It was determined that deployment of the stent was necessary. After we performed PTA using the Gateway MR 2.0 × 12 mm at 4.7 atm for seven seconds, CHIKAI 14 and CHIKAI Extension guidewires (CHIKAI 14, ASAHI Intec, Seto, Aichi, Japan) were used to replace Gateway MR to Excelsior SL-10. Subsequently, the Neuroform Atlas 3.0 × 21 mm (Stryker, Kalamazoo, MI, USA) was placed within the stenosis (Figures 2G-2I). Following this, the ICA was confirmed to be open. Heparin 15,000 units per day was initiated after the procedure.

Postoperative course

**Figure 3:**
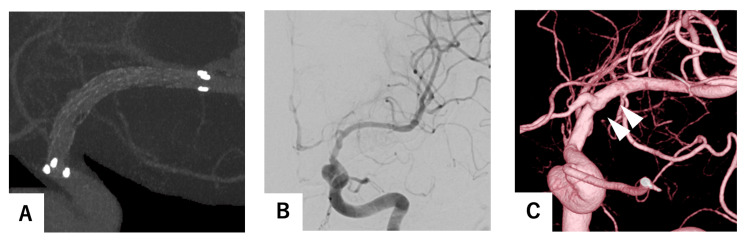
One-year follow-up angiography after the endovascular surgical procedure. (A, B) Cone-beam computed tomography demonstrated adequate stent expansion. (C) Cerebral angiography revealed no stenosis and maintained vascular patency. Additionally, three-dimensional rotational angiography indicated that the stenotic lesion in the internal carotid artery remained dilated.

The patient's aphasia exhibited a notable improvement following the procedure. A continuous heparin regimen was administered for 48 hours following the surgical procedure. The subsequent magnetic resonance imaging (MRI) scan demonstrated that the left ICA was patent (Figure 2K), and DWI did not reveal any enlargement of the infarcted regions (Figure 2J). On the seventh postoperative day, the patient, exhibiting no neurological deficits, was transferred to another medical facility. The patient was maintained on a regimen of 100 mg aspirin and 3.75 mg prasugrel. Rosuvastatin calcium 2.5 mg was administered for dyslipidemia. Amlodipine besilate 5 mg and Telmisartan 40 mg were administered for hypertension. At three months postoperatively, cerebral angiography demonstrated that the left ICA remained open and that the stenosis was dilated. Therefore, aspirin was discontinued and prasugrel 3.75 mg was continued. At the one-year follow-up, cerebral angiography demonstrated optimal patency of the left ICA (Figures 3A-3C). The dilation of the stent was successful, and the vessel lumen was maintained. The dosage of prasugrel was reduced to 2.5 mg.

## Discussion

Achieving recanalization with standard mechanical thrombectomy techniques is challenging in patients with ICAD because the presence of residual stenosis and/or local thrombus formation results in immediate re-occlusion [5].

Prior studies of elective procedures deploying self-expandable stents for symptomatic ICAD have been reported, including the SAMMPRIS trial [6] and the WEAVE study [7]. However, there is a paucity of literature regarding stenting as a salvage technique for AIS patients with ICAD.

PTA and waiting methods represent the initial treatment options for ICAD. The waiting method involves administering antiplatelet medication, deploying the stent retriever into the lesion, waiting a few minutes, and then retrieving it [8]. In the event that these methods do not result in adequate patency, the next step is to consider stenting or bypass.

The use of acute bypass has been demonstrated to be an effective intervention for the treatment of advanced cerebral ischemia when the patient is selected appropriately [9,10]. Nevertheless, such patients are treated with intensified antithrombotic therapy, including rt-PA therapy, which increases the risk of bleeding compared to that observed in typical cases without intensified antithrombotic therapy.

Conversely, stenting for rescue purposes is a more straightforward option than bypass surgery. It can be performed under local anesthesia, can be performed directly after PTA, and can be performed even with intensified antithrombotic therapy. Potential stents include the Wingspan, Neuroform Atlas, and Enterprise [2,11-14].

Wingspan is an open-cell stent indicated for intracranial stenosis. However, because it is an over-the-wire stent, the procedure is more complicated than with other stents. Although the radial force is high, the stent may not be able to adhere to the wall. In the event of re-occlusion, the strut becomes an obstacle, making it difficult to perform additional procedures. Neuroform Atlas and Enterprise are neck-bridge stents for adjunctive treatment of wide-neck aneurysms. These have good wall apposition and a certain degree of radial force, and there have been scattered reports of salvage treatment in ICAD AIS.

Wingspan or Enterprise was placed for salvage in 19 reported cases [11]. All but one of these cases with Enterprise had recanalization equivalent to Thrombolysis in Cerebral Infarction (TICI) 2b or greater. There were no procedural complications, and three patients experienced cerebral hemorrhages. Five patients died within one month, and more than half had a Modified Rankin Scale score of 3 or greater.

The Enterprise stent is easier to deploy than Wingspan because the stent can be placed through a microcatheter with an inner diameter of 0.021 inches. Two reports using the Enterprise stent [13,14] found a total of 14 cases of stent placement, three of which were placed distal to the ICA, as in this case. One had symptomatic intraparenchymal hemorrhage and one had progressive cerebral edema. Both cases resulted in death.

The Neuroform Atlas can be placed through a microcatheter with an inner diameter of 0.0165 or 0.017 inches, and this can increase technical success. Two reports on the implantation of Neuroform Atlas have been published. Yi et al. [2] reported an immediate recanalization rate of 100% with no perioperative complications. Conversely, Al Kasab et al. [12] reported successful implantation in 92.3% of cases, but postoperative symptomatic intracranial bleeding occurred in three patients. The procedural success rate is high, but the long-term durability remains uncertain. In the present case, a Neuroform Atlas stent was implanted, and the patient exhibited a favorable outcome. One year later, the stenotic lesion remained open without restenosis. It has been reported that open-cell stents in extracranial carotid stents cause restenosis less frequently than closed-cell stents [15]. Neuroform Atlas is a low-profile open-cell stent. The good wall apposition and less metal contact with the stenotic lesion may have contributed to the long-term results. On the other hand, if re-occlusion occurs, it is more difficult to secure a true lumen compared to the closed-cell stent of Enterprise, which has disadvantages. It should be noted that this is an off-label use. Further accumulation of reports on long-term outcomes is warranted.

## Conclusions

Neuroform Atlas demonstrated favorable angiographic patency over one year when implanted as a rescue stent in revascularization procedures for ICAD. In comparison to other stents, Neuroform Atlas is easier to implant and safer. Furthermore, its use for rescue purposes in an acute setting may be considered a treatment option.
